# Homology-based inference sets the bar high for protein function prediction

**DOI:** 10.1186/1471-2105-14-S3-S7

**Published:** 2013-02-28

**Authors:** Tobias Hamp, Rebecca Kassner, Stefan Seemayer, Esmeralda Vicedo, Christian Schaefer, Dominik Achten, Florian Auer, Ariane Boehm, Tatjana Braun, Maximilian Hecht, Mark Heron, Peter Hönigschmid, Thomas A Hopf, Stefanie Kaufmann, Michael Kiening, Denis Krompass, Cedric Landerer, Yannick Mahlich, Manfred Roos, Burkhard Rost

**Affiliations:** 1 TUM, Department of Informatics, Bioinformatics & Computational Biology - I12 Boltzmannstr. 3, 85748 Garching/Munich, Germany; 2 Institute of Advanced Study (TUM-IAS) Lichtenbergstr. 2a, 85748 Garching/Munich, Germany; 3 New York Consortium on Membrane Protein Structure (NYCOMPS) & Department of Biochemistry and Molecular Biophysics Columbia University, 701 West, 168th Street, New York, NY 10032, USA

## Abstract

**Background:**

Any method that *de novo *predicts protein function should do better than random. More challenging, it also ought to outperform simple homology-based inference.

**Methods:**

Here, we describe a few methods that predict protein function exclusively through homology. Together, they set the bar or lower limit for future improvements.

**Results and conclusions:**

During the development of these methods, we faced two surprises. Firstly, our most successful implementation for the baseline ranked very high at CAFA1. In fact, our best combination of homology-based methods fared only slightly worse than the top-of-the-line prediction method from the Jones group. Secondly, although the concept of homology-based inference is simple, this work revealed that the precise details of the implementation are crucial: not only did the methods span from top to bottom performers at CAFA, but also the reasons for these differences were unexpected. In this work, we also propose a new rigorous measure to compare predicted and experimental annotations. It puts more emphasis on the details of protein function than the other measures employed by CAFA and may best reflect the expectations of users. Clearly, the definition of proper goals remains one major objective for CAFA.

## Background

### Our contribution to CAFA1

UniProt [1] holds over 22 million sequences (May 2012), but reliable and detailed experimental annotations exist for fewer than 1% of these. GO, the Gene Ontology [2] has become the *gold standard *for function annotation and many methods have emerged that predict GO annotations [3]. Due to various problems, it has been almost impossible to assess how well these methods perform. CAFA (Critical Assessment of Function Annotations) has arisen to address the challenges by a comprehensive independent comparison [4].

CAFA also drove our work presented here. Three teams of students implemented three different methods predicting function through homology, i.e. through inference from experimental annotations of related proteins. All three groups began with the same description what to do, and that description was more comprehensive and detailed than many descriptions of methods in papers. Two of the three methods were surprisingly competitive in CAFA and outperformed other similar methods. This triggered the decision to enhance and combine these classifiers in one meta predictor. This post-CAFA method did NOT participate in CAFA. Would it have, it might have reached the top-10 ranks among all participants. Either way, it suggests several ways for the improvement of function prediction by homology, as demonstrated in this post-CAFA evaluation.

Additionally, we developed a new metric to compare predicted and actual GO annotations. It provides insight into how methods perform with respect to the prediction of the exact functions. This turns out to be largely neglected by existing measures.

### Related work

Several advanced methods have appeared that also predict protein function through homology-based inference. *ConFunc *[5] first assigns the proteins found via PSI-BLAST to groups so that all members of a group share a particular GO term. Looking at the alignments in each group, the method then identifies conserved functional residues, scores them and only outputs the groups above a certain combined score. *GOSLING *[6] first derives various features of the terms found in the BLAST result (e.g. GO evidence code, E-Value and bit score. Using many decision trees, the prediction is then flagged as either correct or incorrect. *PFP *[7] follows an approach very similar to *GOtcha *[8], but also considers highly insignificant BLAST hits and co-occurrence between GO terms. An extension of *GOtcha, ESG *[9], additionally differentiates between the hits found in different PSI-BLAST iterations. *GOstruct *[10] takes the idea of co-occurrence to the next level and builds a sophisticated SVM machinery around "structured output spaces". This refers to the extension of the input space (E-Values, asf.) with all experimentally observed GO-subgraphs. *FANN-GO*[11] uses E-Values as inputs to neural networks. Methods based on data sources other than similarity to already annotated proteins are described in a recent review [3].

## Methods

### GO (Gene Ontology) for CAFA

GO has three parts: Molecular Function Ontology (MFO), Biological Process Ontology (BPO) and Cellular Component Ontology (CCO). CAFA considered only the MFO and BPO. Both correspond to two directed acyclic graphs and capture different aspects of protein function. Functional keywords ("GO terms") are nodes and their relationships are labeled edges. The ontology is hierarchical: following the edges from a node, each new term corresponds to a more general concept of the original function. All paths converge at the root node, which can simply be interpreted as, e.g., *has a molecular function*.

The complete functional annotation of each protein has two subgraphs (MFO and BPO). If a subgraph does not contain all the terms that can be inferred by going from its nodes to the root, we perform a *propagation*. Given a set of GO terms, this operation refers to its extension with all ancestral terms. Ancestrors can be found by following all outgoing paths from the terms to the root: each visited node is an ancestor. If the GO terms have scores (e.g. to reflect their reliability), the latter are also propagated: each parent term is simply assigned the maximum score of its children. Sometimes, a propagated subgraph needs to be reduced again to the *leaf terms*. A leaf term is not the parent of any other term in the propagation and corresponds to *the *most exact description of a function for the given protein.

In order to integrate the operations above into our methods, we used the graph_path table provided by the GO consortium. It contains all possible paths in the entire GO graph, pre-calculated by specific path inference rules.

### Assessment of predicted GO annotations

**Figure 1:**
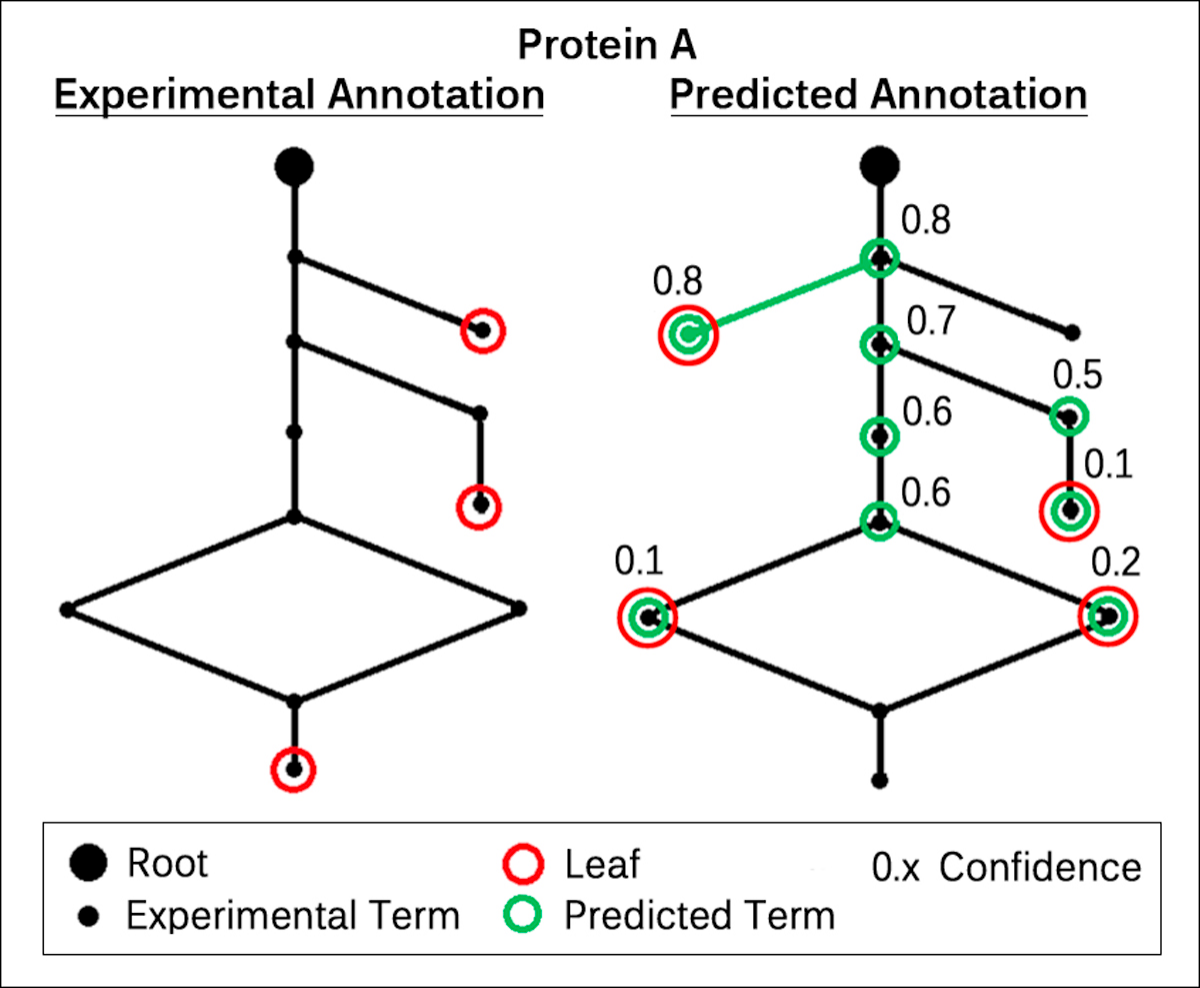
**A functional annotation and its prediction**. This Figure shows one annotation of a sample protein A and its prediction. Each node in a graph corresponds to one GO term and the edges to relationships such as "is a" or "part of". The edges always point to the root node (either "MFO" or "BPO"), which by itself is not informative and discarded in every evaluation. For clearity, the left subgraph only shows the experimental annotation of A. This means, all GO terms have either been experimentally verified or inferred from the same. The red circles indicate the leaf terms, i.e. the nodes which are not a parent of any other term. In the right subgraph, we see the experimental annotation again, but overlaid with predicted terms (green) and their reliabilities. This time, the leaf terms correspond to the predicted GO annotation, instead of the actual annotation.

Analogously to CAFA, we use fixed sets of target proteins to compare prediction methods. Each target corresponds to one or two propagated GO subgraphs of experimentally validated terms (depending on whether both BPO and MFO annotations are available or only one of the two). A method is supposed to predict these subgraphs (e.g. the left tree in Figure 1) and assign a reliability between 0.0 and 1.0 to each predicted term (e.g. green nodes in Figure 1). Then we assess their accuracy in the following ways, separately for the MFO and BPO. For the first two measures, we exclusively used the original CAFA implementations, GO version, targets and target annotations. Only to implement our new leaf threshold measure, we slightly adapted the programs.

#### Top-20

Given the prediction of a single protein, the *top-20 measure *first reduces the prediction to the terms with the highest reliability (Figure 1: green nodes with score 0.8). It then defines recall as the number of correctly predicted GO terms divided by the number of all true GO terms. Precision corresponds to the number of correctly predicted GO terms divided by the number of all predicted GO terms. In Figure 1, for example, recall is 1/11 = 0.09 and precision is 1/2 = 0.5. If a target is not predicted at all, it is assigned a recall of 0.0. Precision is not calculated in such a case and has no influence. Repeating this for all targets, we obtain the average recall and precision. This is the first point in the recall-precision curve. In order to fill the curve, we gradually add the terms with 2nd, 3rd, ..., 20th highest reliability to the predictions and recalculate all of the above.

#### Threshold

The *threshold measure *[4] follows a similar concept as *top-20*. Instead of considering a certain number of terms for each target at a time the measure demands a threshold between 0.0 and 1.0. In case of a threshold of 0.82, for example, each prediction is reduced to terms with a reliability greater than or equal to 0.82. Recall and precision can then be calculated analogously to the *top-20 *measure. A curve is obtained by lowering the threshold in steps of 0.01 from 1.0 to 0.0.

#### Leaf threshold

The *leaf threshold measure*, finally, operates exclusively on the leaves of a propagation (red nodes in Figure 1). First, predicted and experimental subgraphs are reduced to their leaf terms (Figure 1: experimental leaves on the left, predicted leaves on the right). Then, we define a threshold T as before, e.g. T = 0.82, and reduce each prediction to the leaves with a reliability ≥ T. The recall of a single prediction is given by the number of correctly predicted leaves divided by the number of all experimental leaves. Precision is defined analogously. Consequently, we can derive a recall-precision curve in the same way as for the threshold measure. In Figure 1, we obtain the first non-empty prediction as soon as the threshold reaches 0.80 (the highest score of all predicted leaves is 0.8). In this case, recall and precision correspond to 0/3 = 0.0 and 0/1 = 0.0.

The leaf threshold measure is orthogonal to the top-20 and threshold measure: in the case of low recall, for example, the former two measures remove specific GO terms from the prediction and retain only the more general terms. Naturally, more general terms have a higher chance to overlap with the experimental propagation than specific terms, resulting in higher precision. However, the *leaves *of this reduced prediction are not more likely to overlap with the *leaves *of the experimental annotation. If the full prediction was the best estimate of the experimental leaves, the reduced version could even result in recall = precision = 0.0 by the leaf threshold measure, because the reduction might remove all correctly predicted leaves. Our new measure assesses how well the exact functions of a protein are predicted. Too general or specific predictions are penalized.

#### Maximum F1 score

The *top-20 *and threshold measure were the two main metrics in the CAFA meeting. The leaf measure is introduced here for the first time. In order to rank methods, the CAFA organizers additionally used the maximum F1 score over all recall-precision pairs obtained with the threshold measure (*Fmax*). The F1 score is defined as:

(1) $$F_{1}=\frac{2\times precision\times recall}{precision+recall}$$

We also employed Fmax in order to choose among alternative parameters during method development after CAFA.

### Homology-based methods

All the methods that we presented at CAFA were developed as part of the exercises of a regular lecture at the TUM in Munich (year 1-3 in bioinformatics master). Due to limitations in time and resources, our methods had to focus on a k-nearest-neighbor approach and to only use the hits returned from a PSI-BLAST [12] query against Swiss-Prot [1]. They were supposed to optimize the F1 score (threshold measure) of the leaf term with the highest reliability. We split the 16 participating students into three groups, each of which had to develop an own implementation. After 8 weeks of time and one week before the CAFA submission deadline, we received the following three methods. Their key features are summarized in Table 1.

**Table 1:** Comparison of student methods.

	*StudentA*	*StudentB*	*StudentC*
**Input Features**	GO term counts	E-Values	GO term counts; percentage positives

**Scoring Schemes**	1	2	2

**Propagation Types**	maximum child	maximum child	maximum child; cumulative

**Score Normalization Across Proteins**	No	Yes	No

In this table, we have summarized the key differences between student methods. Input features include: the number of times a GO term appeared in the annotations of homologous proteins; the E-Values of the homologous proteins; and the percentage of 'positive' columns in their alignment matrices. Some groups used more than one way to score a GO term or differed during the propagation of a prediction by assigning a node the maximum value of its children or their sum. StudentB normalized the final score of a GO term to improve comparability among proteins.

#### StudentA (Figure 2)

Begin with 2-iteration PSI-BLAST against all Swiss-Prot proteins with GO annotations (E-Value < 0.1). Extract GO terms of the six top PSI-BLAST hits (or all if fewer than 6 hits found). Each identified GO term is scored 1.0 if the term is found in all 6 hits and 0.5 otherwise. Once the term-score pairs have been extracted, only the leaf terms of their propagation are retained. Then apply the following filter to reduce functional redundancy: (i) create *branches *by propagating each predicted leaf term separately; (ii) calculate all pairwise branch overlaps, with the overlap being defined as the number of common GO terms in both branches divided by the average branch size.

**Figure 2:**
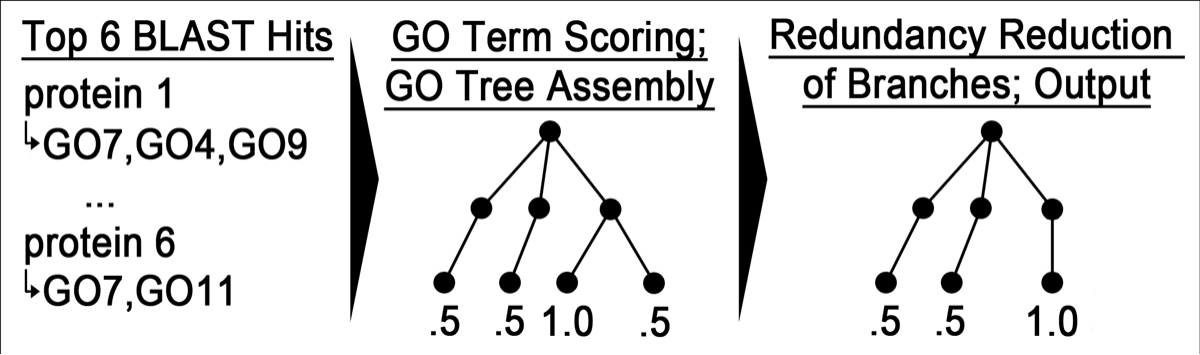
**Flow chart of *StudentA***. *StudentA *first reduces the BLAST output to the best 6 hits. GO terms that are part of the annotation in all 6 hits are assigned a score of 1.0, all others 0.5. Then the predicted GO graph is assembled by propagating the scores and pruned again during a functional redundancy reduction (see text). This reduced graph is output to the user.

Next, cluster all branches so that each pair from two different clusters overlaps less than 10%. For each cluster, the branch with the longest path to the root is chosen, reduced to its original leaf term with the original score and output to the user. As the redundancy reduction may filter out highly supported terms, we apply a final correction: if any pair of branches from previous steps overlaps over 90%, the term common to both and with the longest path to the root, i.e., the lowest common ancestor, is added to the result.

#### StudentB (Figure 3)

Begin with 2-iteration PSI-BLAST against all Swiss-Prot proteins with GO annotations (E-Value < 0.002 for 1^st ^and E-Value < 0.01 for 2^nd ^round). Each PSI-BLAST hit is associated with the propagation of its GO terms and each term in the propagation is associated with the PSI-BLAST E-Value of the hit. We then define two scores.

**Figure 3:**
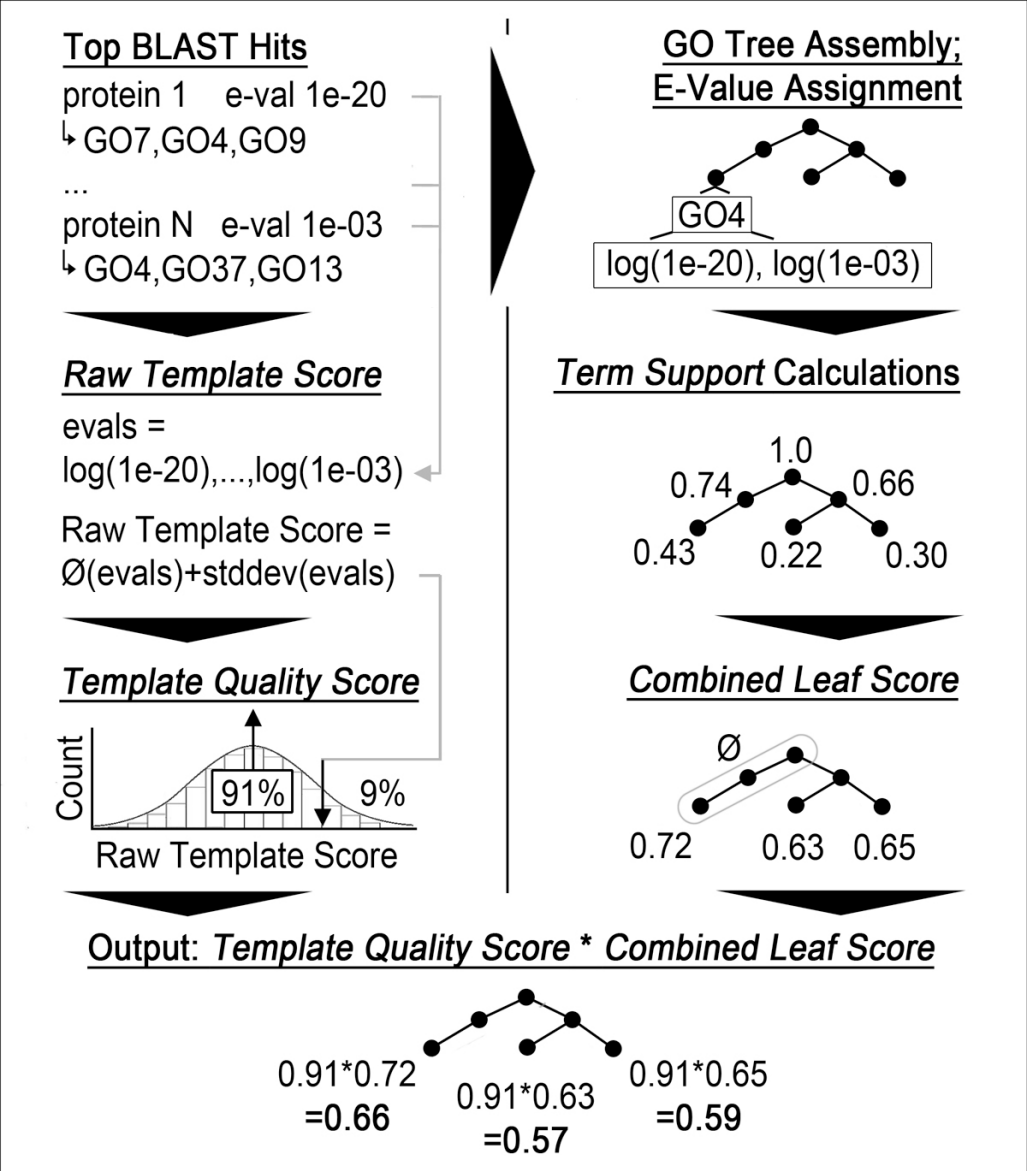
**Flow chart of *StudentB***. *StudentB *first logarithmizes the E-Values of all BLAST hits and averages them. The result is mapped into a range from 0 to 1 by looking up its percentile in a precompiled distribution. This percentile is the *Template Quality Score *and reflects how well we can predict the entire target. To score single terms, we multiply it with the score of each predicted leaf, i.e. the *Combined Leaf Score*. This is the average of the logarithmized E-Values of the nodes on the path from the leaf to the root. The propagation of the leaf terms and their scores is output to the user.

The *template quality score *gauges the reliability of the entire PSI-BLAST query with respect to the goal of assigning function. First, we calculate the *raw template score *as the average over the logarithms of all returned PSI-BLAST E-Values plus twice the standard deviation (also derived from the log(E-Value)). The standard deviation is added to correct for cases with relatively low top scores and relatively high averages. This raw template score is normalized into a value from 0 to 1 by mapping it into a percentile bin obtained from running PSI-BLAST in the same fashion on a sample of all Swiss-Prot proteins (e.g. a score obtained by 90% of the samples for all Swiss-Prot is scored 0.1 = 1-0.9). We call this percentile the template quality score.

The *combined leaf score *measures the reliability of each predicted leaf. First, we compile the propagated set of all GO terms for all PSI-BLAST hits. Each term can occur in multiple hits and thus be associated with multiple E-Values. The *support *of a term is defined as the sum over the logarithm of its E-Values divided by the sum of the logarithm over the E-Values of all hits. The *combined leaf score *of a leaf in the set of GO terms above is then given by the average support of itself and all of its ancestors.

Finally, we multiply *template quality *and *combined leaf *score for each leaf, combine all the leaf-score pairs in one set and output its propagation to the user.

#### StudentC (Figure 4)

Begin with 2-iteration PSI-BLAST against all Swiss-Prot proteins with GO annotations (E-Value < 0.1). Count how often a particular GO term appeared in the PSI-BLAST hits (without propagation). All nodes with counts are propagated through the GO tree. Instead of taking the maximum count of all children at each parent node, however, their values are summed up and added to that of the parent node (normalization to [0,1] by division by maximal value). We call this type of scoring the *max support*. The PSI-BLAST scores, on the other hand, are considered as follows.

**Figure 4:**
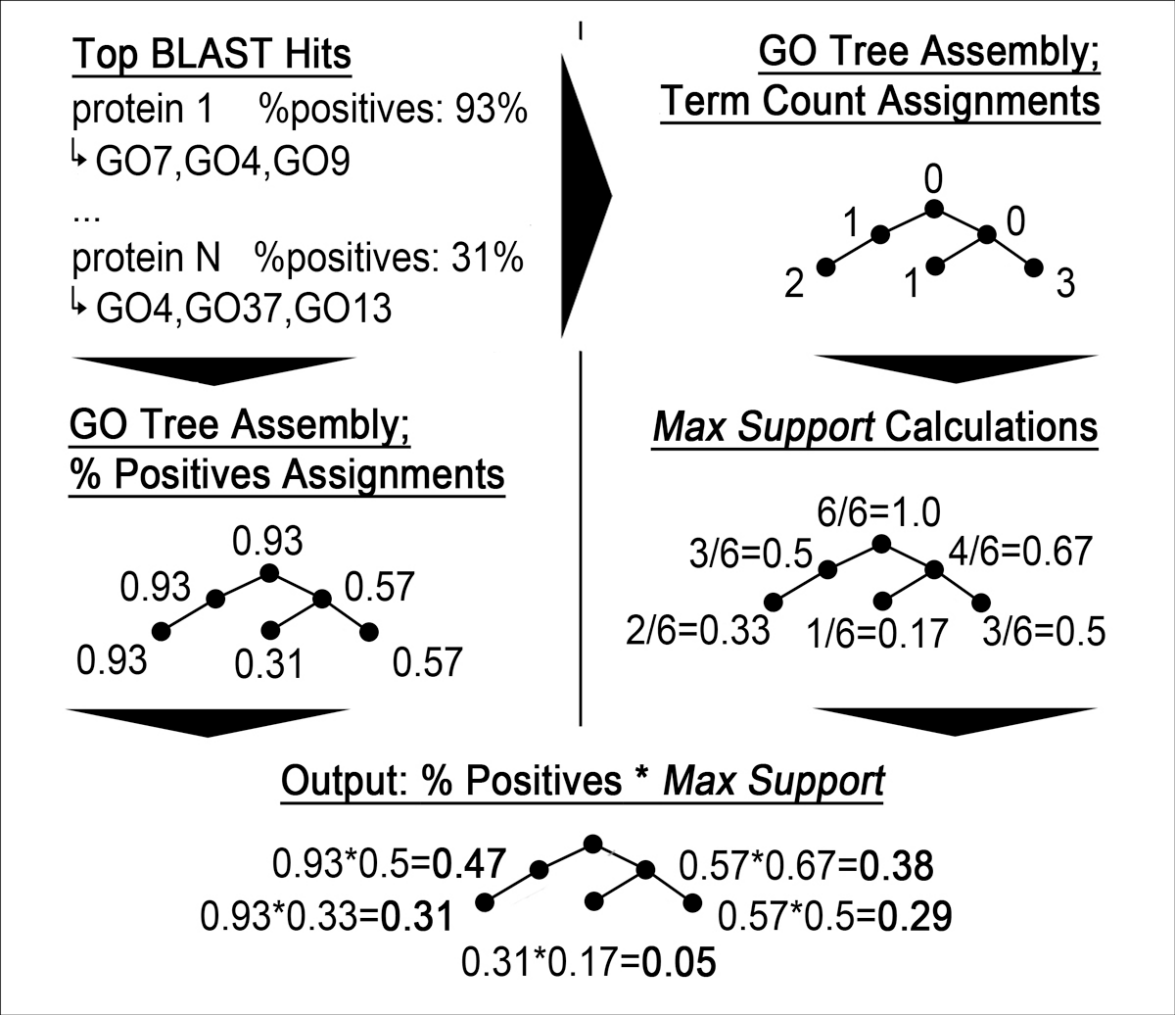
**Flow chart of *StudentC***. *StudentC *first counts how often each GO term appeared in the BLAST hits and performs a cumulative propagation: for each inner node, all counts of its child terms are summed up and added to its own count (depth-first traversal). Dividing each count by that of the root term, we obtain a score between 0 and 1 for each term. In parallel, we calculate a second score for each term by assigning it the maximum percentage of positives of all associated hits (see text). Finally, we multiply the two scores, determine the highest scoring leaf term and output only its propagation to the user.

For each PSI-BLAST hit, we first read off the *positive identity*. This value is included in the default BLAST output and corresponds to the number of *positives *divided by the alignment length. (Each mutation column in the default BLAST output with a positive score by BLOSUM62 is a *positive*.) Then, we multiply the *max support *of each term with the highest associated *positive identity *(we may have many *positive identities*, because a GO term can be associated with multiple PSI-BLAST hits). The method outputs only the one branch corresponding to the highest scoring leaf term.

### Post-CAFA re-parameterization

After CAFA, we parameterized the above three basic homology-inference methods. For *StudentA*, we introduced the options to exclude predictions with a score of 0.5 and to choose the number of PSI-BLAST hits to consider (before: 6; now: 1, 5 or 9). For *StudentC*, we added alternative PSI-BLAST E-Value thresholds (before: 1e-01; now: 1e00, 1e-03 or 1e-06) and percentage pairwise sequence identity as an alternative to the *positive identity*. We also enabled the optional output of all branches, instead of restricting it to the most probable one. The original implementation of *StudentB *had a bug: an alternative graph_path table inverted the order of the columns by mistake. The results of this bug were submitted to CAFA. We fixed the bug and allowed for alternatives in the thresholds for E-Values and maximum numbers of PSI-BLAST hits (E-Value before: 1e-02; now: 1e00, 1e-03 or 1e-06; max. number of hits before: 250 [PSI-BLAST default]; now: 5, 50 or 500).

For all methods, we also add the choice of the number of PSI-BLAST iterations (before: 2 for all methods; now: 1, 2 or 3). Finally, we enabled the filtering out of Swiss-Prot annotations with unclear experimental support (optional restriction to the following experimental GO evidence codes: IDA, IMP, IPI, IGI, IEP, TAS, IC, EXP).

The re-parameterization created 36, 54, and 72 different parameter combinations for *StudentA-C*, respectively. We optimized the parameters by picking the combination leading to the highest Fmax (threshold measure; Eq. 1) on a hold-out data set. This data set comprised all Swiss-Prot proteins annotated with experimentally verified GO terms in 2010 ("Set 2010"). All proteins annotated before 2010 served as templates ("Set < 2010"). This ascertained that there was no overlap to the CAFA targets. In the following, we refer to the optimized student methods as *StudentA'-C'*.

### Post-CAFA method combination

Due to the end of the lecture during which the methods were developed, we could not combine them. We did this also post-CAFA. We randomly split Set 2010 into two equal parts (Set 2010a and 2010b). Parameters were optimized on the first split (2010a; as before, only with 2010a instead of 2010). These optimized variants of *StudentA-C *(say *StudentA''-C''*) were applied to the second split (2010b). Then, we switched the roles of the two sets and repeated the procedure to obtain predictions for each protein in Set 2010. With these predictions, we trained a commonly used meta classifier [13], namely a weighted least-squares linear regression model. This corresponded to the formula x*A' + y*B' + z*C' + i = p, where A', B' and C' are the results of the student methods for each predicted GO term and [x-z] and i are the coefficients to optimize in the regression so that p reflects the reliability of the GO term. In order to meta-predict a new target protein, we first annotate it with methods *StudentA'-C'*. Each predicted GO term is then converted into a vector of three elements (one dimension for each method) and put into the formula above. The resulting value of p is the reliability of the GO term for the given target. We refer to this predictor as *MetaStudent'*.

### Baseline classifiers

The CAFA organizers implemented the following three baseline classifiers to gauge the improvement of current function predictiors over old or naïve methods [11]. (1) *Priors*. Every target has the same annotations and each term's score is the probability of that term occurring in Swiss-Prot. (2) *BLAST*. Target annotations are simply the maximum sequence identity returned by BLAST under default parameters when aligning a target with all proteins annotated with a given term. (3) *GOtcha*. Using the same BLAST results as *BLAST, Gotcha *[8] I-Scores are calculated as the sum of the negative logarithm of the E-Value of the alignment between the target protein and all proteins associated with a given term. Additionally, we introduce *Priors'*, which simply returns the entire GO annotation of a random Swiss-Prot protein. Scores are assigned as in *Priors*.

### Data sets

We used five different data sets for method development and evaluation. All are exclusively derived from GO and the GO annotated proteins from Swiss-Prot and only differ in their release dates. The first three methods used the GO/Swiss-Prot releases from Oct. 2010 ("Set < 2010_10") for both development and group-internal evaluations. We updated to the versions from Dec. 2010 ("Set < 2010_12") and submitted all 48,298 CAFA targets with each method. For post-CAFA developments, we used the release of Jan. 2010 as the source for template annotations ("Set < 2010"). The independent data set needed for post-CAFA parameter optimization then contained all proteins annotated between January and December 2010 ("Set 2010"). Analogously to CAFA, we ignored proteins that had any GO annotation before January 2010 and only retained experimental annotations in the remaining proteins. Experimental GO evidence codes were: IDA, IMP, IPI, IGI, IEP, TAS, IC, and EXP (same as in CAFA). "Set_2010" contained 1752 targets with BPO and 1351 with MFO annotations.

The CAFA organizers provided the original CAFA targets (436 with BPO and 366 with MFO annotations). They correspond to the proteins annotated between January and May 2011 ("Set 2011"). This set was derived following a similar algorithm as those in "Set 2010". The difference was that the CAFA organizers also excluded annotations from the GOA project in proteins annotated before January 2011 (a resource we left untouched). We used the annotations in "Set < 2010_12" to predict proteins in "Set 2011".

Note that this implied that all our post-CAFA optimization could have been accomplished completely BEFORE the submission to CAFA (we just had not been fast enough). Nevertheless, we clearly label all the new methods as "post-CAFA" in this work.

## Results

### Wide performance spread of homology-based inference

**Figure 5:**
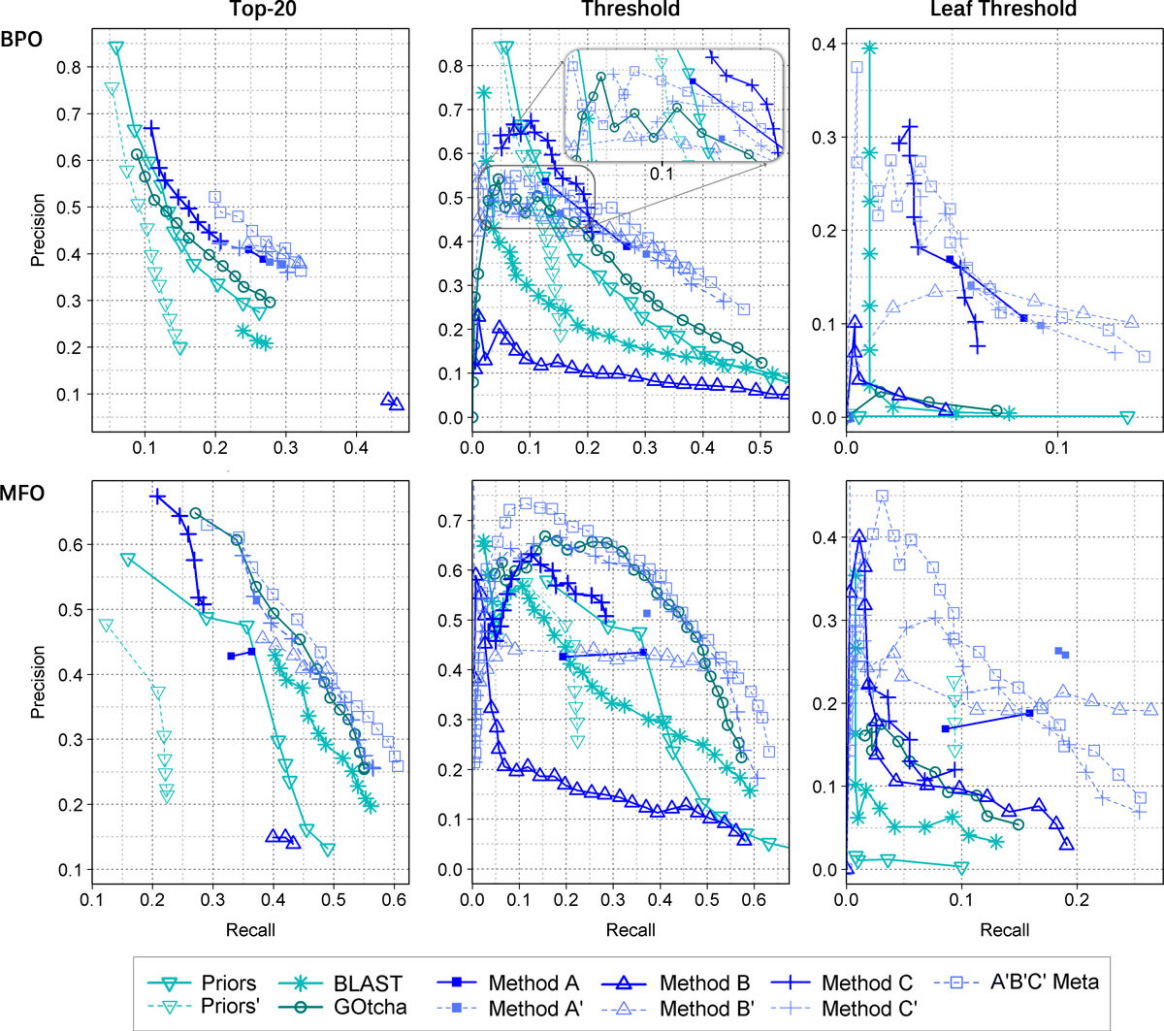
**Results of evaluations before and after CAFA**. Here, we show the results of all methods for each ontology and measure. Baseline classifiers share the same color (cyan), just like methods corresponding to the same design, but different parameter values (blue). Curves derived from the CAFA organizers are solid and bold, otherwise thin and dotted. As the area between recall 0.0 - 0.2 and precision 0.45 - 0.55 is extremely crowded in the BPO threshold measure plot, we provide an enlarged view with the inlet. In the BPO leaf threshold measure plot, *Priors*' is at the origin (0.0, 0.0).

Our three homology-based predictors of protein function (*StudentA-C*) performed very differently (Figure 5, dark blue; note: all data compiled exclusively on the CAFA targets and with data available before the CAFA submission). This was true for both categories, namely for biological process (BPO, Figure 5, top panels) and for molecular function (MFO, Figure 5, lower panels) and for all performance measures (Figure 5: each column signifies one particular measure). For instance, *StudentA *performed slightly better than *StudentC *by the *top-20 *measure (Methods) and slightly worse by the *threshold *criterion (Methods). While *StudentA *and *StudentC *mostly surpassed the baseline tests (*PRIORS *and *BLAST*), they even topped the *GOtcha *baseline (dark green) for many thresholds. In the BPO category (threshold measure), *StudentC *actually outperformed all but two of the other 36 CAFA predictors until a recall of about 0.2 (not shown). Note that the curves for *StudentA-C *in Figure 5 are identical to those calculated by the CAFA organizers.

### Post-CAFA optimization renders homology-based inference competitive

When we changed our methods post-CAFA, we carefully avoided using any information that was not available at the CAFA submission deadline. Nevertheless, we are treating our optimized predictors (*StudentA'-C'*, and *MetaStudent'*) differently to clearly mark the point that these methods did not compete at CAFA. All changes were straightforward (e.g. optimization of simple thresholds) in the sense that they did not require any of the knowledge that we gained at CAFA. They would have been done by anyone with enough time before submission. This reality is important because they improved performance markedly. Our best single method that exclusively used homology information (*StudentC'*) even outperformed the advanced method *GOtcha *in almost all respects. *MetaStudent'*, the combination of all three methods, was consistently on par or better than all others, including *GOtcha*.

### Leaf threshold measure suggested very different view

There is evidence that the top-20 and the threshold measure penalize methods that provide a decision as to which function is predicted and favor methods that output huge lists of scored GO terms (Discussion). If so, their use as the scoring to be optimized may go against the interest of users (Discussion). In contrast, our new *leaf threshold *measure (Methods) favors predictions with reasonable amounts of terms over those with overly many. It first reduces all predicted GO terms to the leaf terms and then compares those to the leaves of the true annotation. Achieving, e.g., a recall of 1.0 simply by outputting the entire GO is therefore impossible. This reveals just how bloated predictions can be (Figure 5: rightmost panels): For instance, the baseline background "method" *Priors *(i.e., predicting all GO terms for each target and scoring them by frequency) is now numerically reduced to where it belongs, namely to a very bad performance. *Priors' *(i.e., randomly picking a protein annotation from Swiss-Prot and scoring terms by frequency), on the other hand, shows up competitive for levels of recall < 0.1 in the MFO category. Since also *Priors *stopped at recall 0.1, there appears to exist a very common low level leaf in GO. In the BPO, a larger and more complex hierarchy, *Priors*' fails, too. It remains unclear whether the bad performance of other baseline classifiers (*BLAST, GOtcha*) under the leaf threshold is due to unnecessarily large predictions in order to achieve high recall or to deeper methodological problems. In any case, our results show that even under this most rigorous measure, we can see fine grained separations between methods.

### Homology-based method ranks very high

CAFA decided to rank methods according to the Fmax score on the threshold measure (Eqn. 1). For compatibility, we followed this approach (Table 2: all methods provided in this contribution, plus the top mark presented at CAFA, namely FunctionSpace). For comparison, we also provide the scores for the top ranking method (FunctionSpace). The complete list presented at CAFA contained 36 methods; for 15 of these, the ranks have been released. Of all methods in Table 2, only *StudentA, Gotcha*, and "best" were in the list. *StudentB-C *were excluded because only one method per group was considered (we had correctly chosen Student A for this). *StudentA'-C' *and M*etaStudent' *were not ranked as they were developed post-CAFA with pre-CAFA data. The best homology-only method (*StudentA*) was in the top 8 only for BPO, while it dropped to rank 13 for MFO. In contrast, two of the baseline methods, namely *Priors *and *Gotcha *both ranked higher for MFO than for BPO. In fact, *StudentA *ranked worse than both baseline methods according to MFO and better than both for BPO.

**Table 2:** Ranking of methods with respect to the maximum F1 score of the threshold measure curves.

	*F1 BPO*	*Rank BPO*	*F1 MFO*	*Rank MFO*
***Presented @ CAFA***				

***GOtcha***	0.29	13	0.47	4

***BLAST***	0.21	-	0.34	-

***Priors***	0.27	15	0.41	12

***StudentA***	0.32	8	0.40	13

*StudentB*	0.15	-	0.20	-

*StudentC*	0.28	14	0.36	-

***Best@CAFA***	0.37	1	0.49	1

***Post-CAFA***				

*Priors'*	0.20	-	0.29	-

*Student A'*	0.33	8	0.43	10

*Student B'*	0.36	3	0.45	7

*Student C'*	0.34	6	0.48	3

*MetaStudent'*	0.36	3	0.48	3

This table shows the maximum F1 score (Fmax) of each threshold measure curve in Figure 5 and its rank in the list of competing methods which was shown at CAFA. This list actually consists of 36 predictors, but only the scores and ranks of the top 15 performers have been released. Classifiers which are actually part of this list are kept in bold. Ranks of other methods are hypothetical, either because calculated after CAFA or because discarded by the CAFA organizers. They considered only one method per participating group and we chose method A. Results for *StudentB *were compiled *with *the bug (Methods).

All our homology-only post-CAFA methods reached F1 scores consistently higher than that of *StudentA*. Our best method (*MetaStudent'*) performed rather well by this criterion and would have ranked in the list of the top three at CAFA, had the method been completed in time. Its F1 scores would have been very similar to those of the top contender (FunctionSpace).

### Ranks varied significantly with measure

We expanded the ranking to include all measures shown in Figure 5 (Table 3). As before, we reduced each recall-precision curve to one maximum F1 score and used this value to define the rank of the respective method for this measure. There were 11 methods, so that ranks always ranged from 1 to 11. Depending on the method, the top and lowest ranks differed by 2 to 7. The average difference for alternative measures was 3.8. Put differently, no single method always had the same rank and, on average, the differences spanned over one third of the entire spectrum.

**Table 3:** Ranking methods by maximal F1 score for various measures.

	*BPO*			*MFO*		
	***Top-20***	***Threshold***	***Leaf***	***Top-20***	***Threshold***	***Leaf***

*Priors*	8	8	11	7	6	11

*Priors'*	10	10	10	10	10	6

*BLAST*	9	9	9	6	9	10

*GOtcha*	6	6	8	2	3	9

*StudentA*	5	5	5	8	7	5

*StudentA'*	3	4	4	5	5	2

*StudentB*	11	11	7	11	11	7

*StudentB'*	2	2	1	3	4	1

*StudentC*	7	7	6	9	8	8

*StudentC'*	4	3	3	4	2	4

*MetaStudent'*	1	1	2	1	1	3

We calculated the maximum F1 score (Eqn. 1) for each method and curve presented in Figure 5 and ranked the methods accordingly. The number in each cell is the rank of the method in the respective category. As we evaluated 11 different methods, ranks range from 1 (best) to 11 (worst). Results for *StudentB *were compiled *with *the bug (Methods).

## Discussion

### A new baseline for simple homology based inference

CAFA provided a common ground to test our student methods with experimental annotations unknown at the time of prediction. These initial methods defined some new lower bounds for the performance of simple homology-based inference (e.g. *StudentA *and *StudentC *for BPO). Our post-CAFA optimizations were carried out exclusively with data available before the CAFA submission deadline. Hence, we postulate that our new results could have been presented at CAFA, had we been ready in time. They show that simple homology based inference can compete with state-of-the-art prediction methods. Considering the wealth of data that we did not use, this suggests large room for improvement.

### Similar methods can differ substantially

Nearest neighbor based homology inference can be realized in surprisingly many ways. The details of an implementation may lead to almost random predictions (*StudentB, BLAST*) or to state-of-the-art tools (optimized student methods, *GOtcha*). This pertains to both design choices and other free parameters. For example, score normalization across targets appeared deleterious for low-recall precision (*StudentB'*). In contrast, restricting predictions to the most probable leaf can boost this aspect of performance (*StudentC*). Analyzing the impact of various free parameters during the optimization process (data not shown), we also found the choice between using all or only experimental GO annotations to be critical: *StudentA' *and *StudentC' *only approached the performance of *Gotcha *in the MFO category because they focused on experimental annotations.

### CAFA measures seem to favor unspecific predictions

The difference in the performance of *Priors *and *Priors' *for the top-20 and threshold measures must be the result of *Priors' *reducing predictions to observed protein annotations. Both predictors use the same scoring (term frequencies). To understand this effect, consider a minimal scoring threshold of 0.1. This defines one point in a recall precision curve. Any term with a frequency of, e.g., 0.15 will be a correct prediction for about 15% of the targets with *Priors*, because this predictor always predicts the entire GO ontology. In contrast, *Priors' *will pick this term in only about 15% of all cases, reducing the chance to predict it correctly to 0.15*0.15 = 0.02 = 2%. Put simply, *Priors *finds many more true positives than *Priors' *at any reasonable threshold. Superfluous predictions, on the other hand, should be more frequent for *Priors *than for *Priors'*, because *Priors *always predicts all terms above 0.1 and *Priors' *only a fraction. Depending on which of those two error forces is stronger, either *Priors *or *Priors' *is preferable for *top-20 *and *threshold*. At least for the GO and typical Swiss-Prot annotations, the incorporation of many false positives in the prediction seems highly favorable (*Priors*). We would be surprised if this effect was limited to *Priors *and *Priors' *and not observable for most other scoring schemes.

### The *leaf threshold *measure challenges existing metrics

The leaf threshold measure sheds a brighter light onto many results presented at CAFA. Now, baseline classifiers are hugely outperformed by, for instance, homology-based methods and also a different type of random predictor is favorable (*Priors'*). This can be explained by a simple example: assume the propagated annotation of a single-function aldolase enzyme contains four terms. Predicting it, we obtain, e g., a subgraph of 20 terms in which the four highest scoring terms are correct and the others are wrong According to the *top-20 *and *threshold *measure, this is very good: We reach a recall of 1.0 with a precision of 1.0 (first four terms). Only when considering all predictions, precision drops to 0.2 (4/[4+16]).

We argue that this type of measure does not reflect performance from a user's point of view. Using all predicted terms, he or she will end up with the precision of the highest recall. Even considering the predicted reliability of each term, the user still has to decide which terms are correct and which are not: the reliability threshold separating true from false terms is unknown. The odds of choosing a threshold exactly between the score of the 4th term (correct) and the 5th term (false) are low. But exactly this choice is assumed by the *top-20 *and *threshold *measures and can highly bias exact function prediction, as evident in the results of the leaf threshold measure. The latter yields a recall and precision of 1.0 only if the prediction solely consists of the first four highest scoring terms. Note that also restricting the prediction to scores above a certain threshold per default does not solve the problem: first of all, we would have to find this global threshold that leads to the best leaf accuracy (a minimal change of the threshold can lead to entirely new leaves and a new number of leaves). This, however, should clearly be the task of the method developer, not of the assessor. Secondly, even with the best threshold, a small internal change of the method might still lead to better performance. For instance, consider a variable threshold that depends on the target (instead of one global threshold) or a restriction of the output to terms that have already been observed as leaves in Swiss-Prot. Again, this is the task of the method developer, not of the assessors.

We are convinced that the *leaf threshold *measure will be an important extension for CAFA2. Getting to points such as 80% recall at 10% precision with the current measures is really not a valid goal for function prediction. Rather the opposite, best performance should imply high accuracy/precision. This direction is supported by our new measure.

### Method ranking might be misleading

Our ranking of methods (Tables 2 and 3) followed guidelines proposed earlier [14-16]. For example, we always used the same data set and scoring schemes for all methods. However, no clear "winner" emerged, as it depended on the measure which method ranked top. *MetaStudent'*, for instance, performed best on average, but ranked only 3^rd ^behind *StudentB' *and *StudentA *by *the leaf threshold *measure in the MFO category (Table 3). In addition, there are many alternative relevant performance measures and many new methods are yet to be published. For future CAFA experiments, it will therefore become even more important to avoid "crowning winners" (unless methods stand out by all means) and to focus on method groups suited best for certain disciplines.

## Conclusions

In this work, we have explored the design and parameter space of homology-based function prediction based on nearest-neighbor principles. We find that small methodological and parametric changes can cause dramatic differences in performance. Consequently, we propose several new algorithms that outperformed similar methods either at the CAFA meeting or in the assessment presented here. Consistently showing superior accuracies, our best predictor even imposes itself as a substitution of the popular *Gotcha *method suggesting that a loose coupling of diverse nearest-neighbor methods can yield state-of-the-art performance. Finally, we challenge existing evaluation protocols. Apparently, the performance measures on which CAFA focused inadequately encourage methods to abstain from making specific function predictions and to instead provide huge lists of scored GO terms. This appears a push into the wrong direction. Therefore, we introduced a new rigorous measure that corrects for this shortcoming as a candidate for the assessment at CAFA2.

## Availability

Project name: Metatstudent

Project home page: https://rostlab.org/owiki/index.php/Metastudent

Operating systems: Unix

Programming languages: Python, Java, Perl

Other requirements: BLASTP

License: GNU GPL for academic user; commercial licence otherwise.

## Authors' contributions

TH was one of the supervisors of the exercises, developed and implemented methods after CAFA, carried out the evaluations and drafted the manuscript. RK and SS were two of the students who developed and implemented methods Student A-C and additionally volunteered to help implementing Priors'. EV and CS supervised the exercises. DA, FA, AB, TB, MHec, MHer, PH, TH, SK, MK, DK, CL, YM and MR developed and implemented methods Student A-C. BR had the initiating ideas of this work and majorly revised the drafts of the manuscript.

## Competing interests

The authors declare that they have no competing interests.

## Declarations

This article has been published as part of *BMC Bioinformatics *Volume 14 Supplement 3, 2013: Proceedings of Automated Function Prediction SIG 2011 featuring the CAFA Challenge: Critical Assessment of Function Annotations. The full contents of the supplement are available online at http://www.biomedcentral.com/bmcbioinformatics/supplements/14/S3.
